# Antimicrobial Activity of Quinazolin Derivatives of 1,2-Di(quinazolin-4-yl)diselane against Mycobacteria

**DOI:** 10.1155/2017/5791781

**Published:** 2017-05-22

**Authors:** Bikui Tang, Meili Wei, Qun Niu, Yinjiu Huang, Shuo Ru, Xiaofen Liu, Lin Shen, Qiang Fang

**Affiliations:** ^1^School of Life Science, Institute of Neurobiology, Anhui Key Laboratory of Infection and Immunity, Bengbu Medical College, Bengbu 233030, China; ^2^Department of Physiology, Bengbu Medical College, Bengbu 233030, China; ^3^Scientific Research Center, Bengbu Medical College, Bengbu 233030, China; ^4^Department of Microbiology and Parasitology, Anhui Key Laboratory of Infection and Immunity, Bengbu Medical College, Bengbu 233030, China

## Abstract

*Mycobacterium tuberculosis (M. tuberculosis)* is one of the leading causes of morbidity and mortality. Currently, the emergence of drug resistance has an urgent need for new drugs. In previous study, we found that 1,2-di(quinazolin-4-yl)diselane (DQYD), a quinazoline derivative, has anticancer activities against many cancers. However, whether DQYD has the activity of antimycobacterium is still little known. Here our results show that DQYD has a similar value of the minimum inhibitory concentration with clinical drugs against mycobacteria and also has the ability of bacteriostatic activity with dose-dependent and time-dependent manner. Furthermore, the activities of DQYD against* M. tuberculosis* are associated with intracellular ATP homeostasis. Meanwhile, mycobacterium DNA damage level was increased after DQYD treatment. But there was no correlation between survival of mycobacteria in the presence of DQYD and intercellular reactive oxygen species. This study enlightens the possible benefits of quinazoline derivatives as potential antimycobacterium compounds and furtherly suggests a new strategy to develop new methods for searching antituberculosis drugs.

## 1. Introduction

*Mycobacterium tuberculosis* is a serious pathogen worldwide, which infects one-third of the world's population. WHO newest reporter shows that about 10.4 million people developed tuberculosis and 1.4 million people die from the disease in 2015 [1].

Therapy of tuberculosis is using chemotherapy compounds, which categorize as the first- and second-line drugs. Currently, the first-line antituberculosis drugs include rifampicin, streptomycin, isoniazid, pyrazinamide, and ethambutol. The second-line drugs are kanamycin, ciprofloxacin, ethionamide, and so on. In clinical, the treatment of tuberculosis is a long-duration and multidrug treatment plan, which needs at least four drugs used together for six months [2]. However, unregulated and timeless use of antituberculosis drugs causes serious resistance. Nowadays, the emergence of multidrug-resistant (MDR-TB) and extensively drug-resistant TB (XDR-TB) urgently calls for the development of drug with new targets and mechanism [3].

Unfortunately, there are few new clinical drugs developed against* M. tuberculosis* in past several decades, although researchers found various potential antituberculosis compounds, such as quinoline, quinolone, and fluoroquinolone. Bedaquiline, a novel mycobacterium-specific ATP synthase inhibitor, is the first drug to be approved for the treatment of MDR-TB in the last 40 years [4]. Meanwhile, some of these compounds were undergoing clinical trials, such as moxifloxacin and TMC207 [2]. Novel imidazoline antimicrobial scaffold inhibits DNA replication with activity against* Mycobacteria *[5]. Teixobactin, a new antibiotic from uncultured bacteria, showed high activity against* M. tuberculosis* H37Rv through inhibiting cell wall synthesis by binding to a highly conserved motif of lipids II and III [6].

In previous study, we found that several quinazoline derivatives have anticancer activities. 1,2-Di(quinazolin-4-yl)diselane (DQYD) was synthesized through introducing selenium into quinazoline lead structure and exhibited considerable antitumor activities in many cancer cell lines, through inducing cell apoptosis and membrane damage [7–9]. Meanwhile, our colleague also found that DQYD has inhibiting effect on toxoplasma in vitro [10] and displays a potential ability to inhibit pathogens of plants (unpublished data). All these results implied that DQYD had general inhibition activities against both eukaryote and prokaryote.

Based on these observations, we investigated the effect of DQYD against mycobacteria in this study. Our results show that DQYD inhibits* M. smegmatis* and* M. tuberculosis* growth with low value of MIC in vitro. Furthermore, DQYD results in dysregulation of intercellular ATP homeostasis and increased mycobacterium DNA damage. This study revealed that DQYD was a potential antimicrobial compound and furtherly suggested a new strategy to develop antituberculosis drugs.

## 2. Material and Methods

### 2.1. Bacterial Strains and Culture

The* Mycobacterium smegmatis (M. smegmatis)* mc^2^155 strains were cultured in 7H9 broth supplemented with 0.2% (v/v) glycerol and 0.05% (v/v) Tween-80 (7H9) or on Luria-Bertani agar supplemented with 0.5% (v/v) glycerol (LBG). The* Mycobacterium tuberculosis (M. tuberculosis) *H37Ra and* Mycobacterium bovis (M. bovis)* BCG were grown in 7H9 broth supplemented with 0.2% glycerol, 0.05% Tween-80, and 10% OADC (7H9-OADC) or on 7H11 plates supplemented with 0.5% glycerol and 10% OADC (7H11-OADC). The* Escherichia coli (E. coli)* MG1655 were cultured in LB broth and LB agar. All strains were determined and stocked in our laboratory. The 7H9 broth, 7H11 broth, and OADC were purchased from BD Difco. The Luria-Bertani and agar were bought from Oxford.

### 2.2. Compound of 1,2-Di(quinazolin-4-yl)diselane

**Figure 1:**
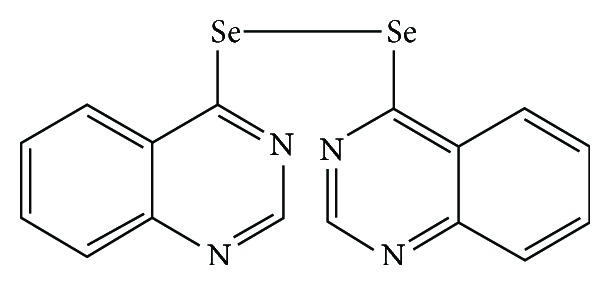
The chemical structure of 1,2-di(quinazolin-4-yl)diselane.

1,2-Di(quinazolin-4-yl)diselane (DQYD) was synthesized by our college, which has two-quinazolin structure and a diselane perssad (Figure 1) [11]. In this study, DQYD powder was dissolved in DMSO solution (Sigma) to storage concentration of 50 mg/mL (125 mM) at −20°C and then dissolved in phosphate buffer saline (PBS) to working concentration before being used, avoiding the effects of DMSO in experiments.

### 2.3. MIC Determined

The minimum inhibitory concentration (MIC) was the lowest concentration that prevented bacteria visible growth. The MIC of DQYD was determined by using serial 2-fold dilutions of the compound in medium. Bacterial cultures at exponential phase (OD_600_ = 0.6~0.9) were diluted to 1 × 10^6^ cells/mL and exposed to different concentrations of DQYD in 96-well plates at 37°C for 3 days (for* E. coli*), 7 days (for* M. smegmatis*), and 21 days (for* M. tuberculosis* and* M. bovis*).

### 2.4. Killing Kinetic Curves Assay

To obtain killing kinetic curves of DQYD against mycobacteria,* M. smegmatis* mc^2^155 and* M. tuberculosis* H37Ra cultures at exponential phase were treated with different concentration of DQYD (within 1~50 × MIC) over the course of 4 weeks [12]. Aliquots of cultures were removed at indicated time points and plated for the colony forming units (CFUs) count.

### 2.5. Quantification of Intracellular ATP Concentration

To gain insight into the ATP levels in DQYD against mycobacteria, we monitored the ATP concentration over a 4-week course. Intracellular ATP was extracted with acid method. Briefly, bacterial cells from 1 mL of culture were lysed in 100 *μ*L 0.1 M HCl by heating at 65°C for 10 min and then neutralized with 100 *μ*L 0.1 M NaOH. The aliquots were collected by being centrifuged with 12,000*g* for 5 minutes. The amounts of intracellular ATP were quantified by using the BacTiter-Glo Microbial Cell Viability Assay Kit (Promega). 10 *μ*L of cell lysates was transferred into 96-well blank plates and added to 100 *μ*l with PBS and then mixed with an equal volume of the BacTiter-Glo reagent and incubated for 5 min in the dark. The emitted luminescence was detected using GloMax 96 Microplate Luminometer (Promega) and expressed as relative luminescence density. The ATP standard was an internal control which was ranged from 0.1 to 100 nM.

### 2.6. TUNEL Assay

To investigate the bacterial genomic DNA damage during DQYD treatment, terminal deoxynucleotidyl transferase-mediated dUTP-biotin nick end labelling (TUNEL) was used. Mycobacterium cultures were washed once with 1 mL cold PBS at indicated time points. Cells were fixed with 1 mL 2% (w/v) paraformaldehyde and then incubated on ice for 60 minutes. Cells were washed once in 1 mL cold PBS and suspended in 1 mL ice cold 70% ethanol. DSBs (double stands breaks) were labeled by FITC-dUTP for staining and propidium iodine (PI) as a counterstain (APO-DIRECT Kit, BD). For staining, the washed cells were suspended in 50 *μ*L 2% SDS and then washed again. Labeling was performed by suspending cells in 50 *μ*L DNA labeling solution for 3 h at 37°C. Cells were washed twice with the rinse buffer and suspended in 0.5 mL PI/RNase staining buffer. Stained cells were analyzed by BD FACS Verse. TUNEL positive cells were identified using FlowJo software and reflected the number of FITC-dUTP positive cells in all PI positive cells.

### 2.7. ROS Assay

To investigate the role of reactive oxygen species (ROS) in antimicrobial activity of DQYD, approximately 1 × 10^6^* M. tuberculosis* H37Ra cultures cells treated with DQYD were collected at the indicated time points; then the cells were washed twice with PBS and stained with dihydroethidium (Invitrogen). Cells were immediately detected using FACS Verse (BD) and analyzed by FlowJo software.

### 2.8. Iron Concentrations Assay

*M. tuberculosis* H37Ra cultures treated with DQYD were harvested by centrifugation. The cell pellets were washed twice with ice-cold PBS, resuspended with glass beads, and lysed using Mini-Beadbeater. The total iron, ferrous iron, and ferric concentration were detected according to the instruction of Iron Assay Kit (Sigma). Briefly, culture samples were rapidly homogenized in 4~10 volumes of cold iron assay buffer and centrifuged at 12,000*g* for 10 minutes at 4°C to remove insoluble material. Then 5 *μ*L of iron assay buffer or iron reducer was added to the sample and mixed well. Then the reaction is incubated for 30 minutes at room temperature. Add 100 *μ*L of iron probe to each well and incubate the reaction for 60 minutes. Finally, iron concentration was measured at the absorbance at 593 nm and calculated according to the instruction.

### 2.9. Statistical Analysis

Statistical significance was analyzed with the unpaired two tailed Student's *t*-test using GraphPad Prism 7.0 software.

## 3. Results and Discussion

### 3.1. MIC of DQYD against Microbe

To estimate the potential antibacterial activity of DQYD, the MIC was measured firstly. As mentioned in Material and Methods,* E. coli, M. smegmatis, M. bovis, *and* M. tuberculosis* strains were cultured to Log phase, and 1 × 10^6^ cells were exposed to the corresponding medium containing serial 2-fold dilutions DQYD for indicated times. As shown in Table 1, the MIC values were 4 *μ*g/mL (10 *μ*M), 2 *μ*g/mL (5 *μ*M), and 1 *μ*g/mL (2.5 *μ*M) for* M. smegmatis* mc^2^155,* M. bovis* BCG, and* M. tuberculosis *H37Ra, respectively, while the value exceeded 256 *μ*g/mL for* E. coli* MG1655 strain. Meanwhile, the MIC of two first-line drugs, rifampicin and streptomycin, were also determined. The values of rifampicin were 2, 0.125 and 0.5 *μ*g/mL for* M. smegmatis, M. bovis,* and* M. tuberculosis*, respectively; meanwhile, the results of streptomycin were 4, 0.25, and 0.5 *μ*g/mL. These data implied that DQYD had potential ability to inhibit mycobacteria growth with lower concentration, similar to the antituberculosis drugs, rifampicin and streptomycin. However, DQYD has no effect on* E. coli*. Considering these results and biosafety reasons, we investigated the mechanisms of DQYD against* M. tuberculosis* H37Ra in the following study.

### 3.2. Killing Kinetic Curves of DQYD against* Mycobacterium*

**Figure 2:**
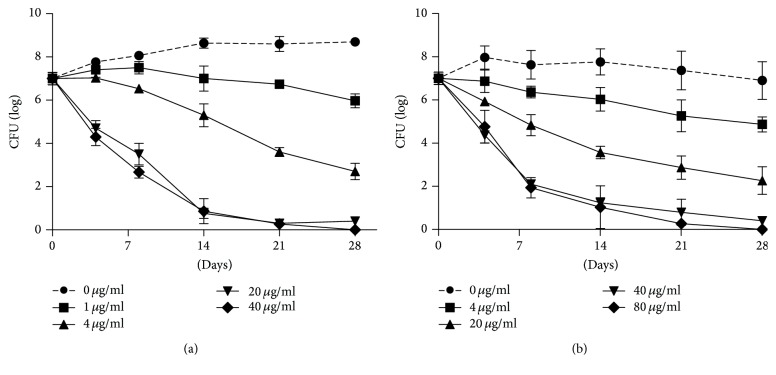
Killing kinetic curves of DQYD against mycobacteria.* M. tuberculosis *H37Ra (a) and* M. smegmatis* MC^2^155 (b) cultures grown to exponential phase were diluted to 1/20 and treated with different amounts of DQYD (from 0 to 80 *μ*g/mL) for indicated time points. CFUs were determined by plating tenfold serial dilutions and incubating the plates at 37°C for 4 days (for* M. smegmatis*) or 21 days (for* M. tuberculosis*).

According to the above results, killing kinetic curves of DQYD against* M. tuberculosis *were measured. H37Ra cultures were treated with different concentrations of DQYD and plated for CFUs counting over the course of 4 weeks. We find that a dose-dependent manner was observed and an obvious inhibition was obtained at 20 *μ*g/mL DQYD treatment. Strikingly, 40 *μ*g/mL and 80 *μ*g/mL DQYD inhibited mycobacteria cultures in 8–12 days. In time analysis, treatment of log-phase* M. tuberculosis* at 40 *μ*g/mL resulted in 2.2-log and 5-log reduction in viable bacteria after 4 days and 8 days, respectively, indicating that DQYD has bacteriostatic activity in vitro (Figure 2(a)). Meanwhile, killing kinetic curves of DQYD against* M. smegmatis *MC^2^155* strain has *shown similar result (Figure 2(b)).

### 3.3. Intracellular ATP Level of Mycobacteria during DQYD Treatment

ATP is catalyzed by ATP synthase and produced through the electron transport respiratory chain. Recently, bacterial energy metabolism has become a new target for the antimycobacterial drugs development [13, 14]. For example, TMC207 is a new clinical diarylquinoline drug candidate, which blocks the ATP synthase and has an efficient treatment of* M. tuberculosis *[15, 16].

**Figure 3:**
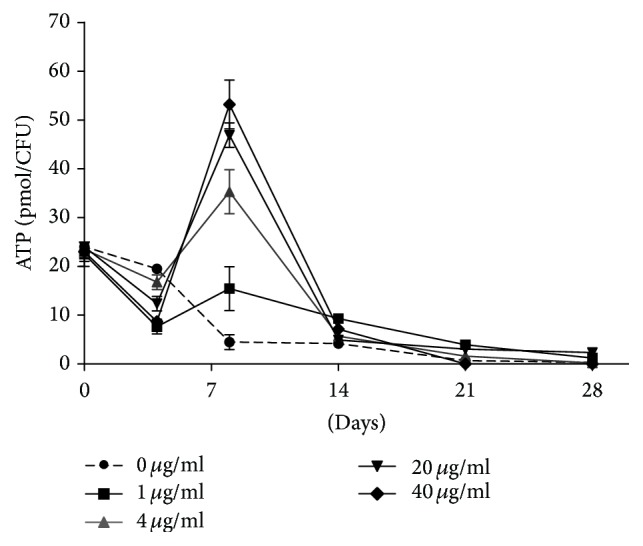
Intracellular ATP levels during DQYD treatment.* M. tuberculosis *H37Ra cultures grown to exponential phase were diluted and treated with DQYD (from 0 to 40 *μ*g/mL). At indicated times, the CFUs were determined by plating tenfold serial dilutions and incubating the plates at 37°C for 21 days and the intracellular ATP concentration was measured by the BacTiter-Glo Microbial Cell Viability Assay. The ATP level was expressed as pmol/CFUs (1 × 10^8^ cells).

To address the energetic status in mycobacteria during DQYD treatment, we measured the intracellular ATP level over a 4-week course. We found that the ATP concentration in* M. tuberculosis* H37Ra cultures (0 *μ*g/mL) progressively reduced and reached the minimal level at about 2-3 weeks and then remained constant later. The cultures treated with DQYD also exhibited a decline of ATP level after 2 weeks, similar to the untreated group. However, the ATP level was significantly higher than that of 0 *μ*M during 1-2 weeks, especially in 8 days, consistent with the analysis of killing kinetic curves. Interestingly, the increased level of ATP had the dose-dependent manner of DQYD (Figure 3). These observations indicated that intracellular ATP level played roles in sterilizing mycobacteria cultures by DQYD [17]. These results show that a reduced ATP level is maintained in growth stationary phase of mycobacteria and furtherly imply that de novo ATP homeostasis is required for the maintenance of* M. tuberculosis* survival. Furthermore, antituberculosis drugs may have ability to break this homeostasis and result in inhibit the mycobacteria.

### 3.4. DNA Damage in* Mycobacterium *Treated with DQYD

Discontinuity of the chromosome is a lethal reason in microorganisms because it interrupts genomic replication and stability [18]. Double-strand DNA breaks (DSBs), a discontinuity of the chromosome, are the most cytotoxic form to result in DNA damage [19]. To investigate whether DQYD against mycobacteria could be a consequence of DNA damage, DSBs level was labeled with TUNEL methods and measured in* M. tuberculosis *H37Ra cultures using flow cytometry at indicated time points.

**Figure 4:**
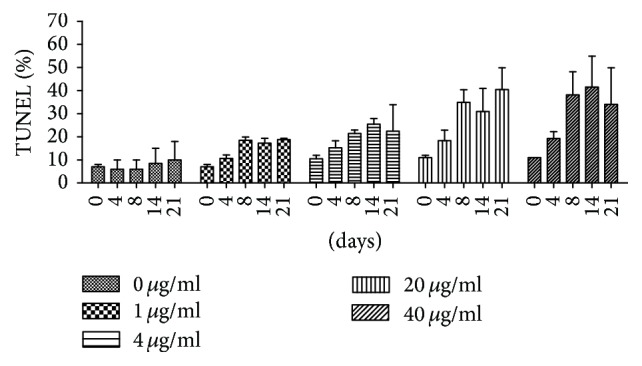
TUNEL level after DQYD treatment in time-dependent manner.* M. tuberculosis *H37Ra cultures grown to exponential phase treated with various amounts of DQYD (from 0 to 40 *μ*g/mL). At indicated times, the cells were labeled by FITC-dUTP/PI and measured by flow cytometry.

**Figure 5:**
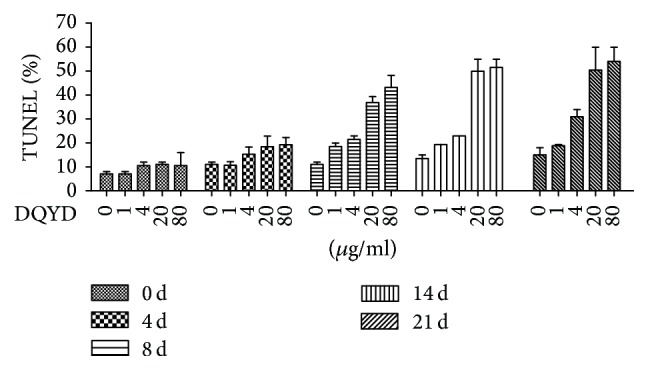
TUNEL level after DQYD treatment in dose-dependent manner.* M. tuberculosis *H37Ra cultures grown to exponential phase treated with various amounts of DQYD (from 0 to 40 *μ*g/mL). At indicated times, the cells were labeled by FITC-dUTP/PI and measured by flow cytometry.

We found that the TUNEL level was strikingly increased after DQYD treatment (from 0 to 40 *μ*g/mL). As shown in Figure 4, obvious change was not observed in untreated group (0 *μ*g/mL) over 21-day course, but there were about 2–4-fold increase after DQYD treatment for 8 days compared to 0 days in different concentration group, and this trend became more significant in 14 and 21 days, implying that the increase of TUNEL level was dependent on the time course. Meanwhile, we found that TUNEL level was also related to the DQYD concentration (Figure 5). There was about at least 4-fold change in 20 *μ*g/mL and 40 *μ*g/mL compared to 0 *μ*g/mL after 8 days. All these results have shown that the TUNEL was increased by DQYD with a dose-dependent and time-dependent manner, implying that DNA damage underlies killing mycobacteria by DQYD and is consistent with the results of killing kinetic curves.

### 3.5. ROS and Iron in* Mycobacteria *Treated with DQYD

Recently, a common mechanism of killing by bactericidal drugs has been reported, which involves the generation of reactive oxygen species (ROS, including superoxide, hydrogen peroxide, and hydroxyl radicals) by the Fenton reaction and results in DNA damage in bacteria including* M. tuberculosis* [12, 20]. However, this mechanism was challenged by other researchers, who declared that killing or cell death by bactericidal antibiotics does not depend on ROS [21, 22].

**Figure 6:**
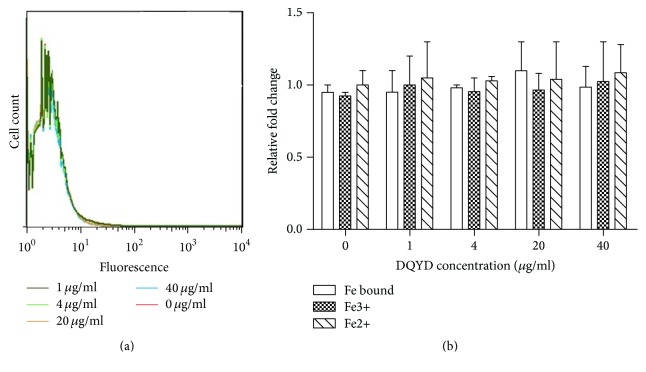
ROS levels and iron concentration during DQYD treatment.* M. tuberculosis *H37Ra cells treated with different concentrations of DQYD (0-40 *μ*g/mL) for 14 days (a) and then stained with dihydroethidium for ROS detection. The ROS levels were measured by flow cytometry and the data was analyzed using FlowJo. Total iron bound and ferrous and ferric iron concentrations in* M. tuberculosis* H37Ra treated with DQYD for 14 days were measured as described in Material and Methods (b).

To test whether DQYD activity against mycobacteria could be a consequence of this mechanism, ROS levels were measured in* M. tuberculosis* H37Ra. At the indicated time points, the cultural cells were stained with dihydroethidium and detected using flow cytometry. We found that there was essentially no difference in survival of* M. tuberculosis* treated with DQYD for 8 and 14 days, implying that ROS may not play a role in killing of mycobacteria (Figure 6(a)). Moreover, ROS levels of all groups were of low value; this is in part because of the sensitivity and detection of staining.

Furthermore, the generation of ROS was produced by irons via Fenton reactions. To examine the role of iron level in antimicrobial activity of DQYD, the concentrations of bound iron and reduced and oxidized free iron were measured in* M. tuberculosis* H37Ra cultures. Similar to the results of ROS, intracellular bound iron and free iron (ferrous and ferric) levels were not changed after DQYD treatment for 8 and 14 days (Figure 6(b)).

The results of ROS and iron level implied that oxidation/reduction mechanisms might not be involved in antimicrobial activity of DQYD, suggesting that the mechanisms of DNA damage need further investigation.

## 4. Conclusion

In summary, this study indicates that 1,2-di(quinazolin-4-yl)diselane (DQYD), a quinazoline derivative, is a potential antimycobacterium compound. DQYD has a low value concentration of MIC and bactericidal activity in a dose-dependent manner, which inhibits mycobacteria cultures in 8–12 days. Furthermore, the activity of DQYD against* M. tuberculosis* is associated with intracellular ATP homeostasis and DNA damage level. Meanwhile, there was no correlation between mycobacteria survival in the presence of DQYD and intercellular level of ROS and iron level. All of the results suggest that DQYD may be a new antimycobacterium potential drug with bacteriostatic activity, but the intrinsic mechanisms need further investigation. This study also provides approach to develop new methods for searching antituberculosis drugs from other research fields.

## Figures and Tables

**Table 1:** MIC of DQYD against microbe.

	MIC (*μ*g/mL)			
	*M. smegmatis* mc^2^155	*M. bovis* BCG	*E. coli* MG1655	*M. tuberculosis* H37Ra
DQYD	4	2	>256	1
Rifampicin	2	0.125	—^*∗*^	0.5
Streptomycin	4	0.25	—^*∗*^	0.5

^*∗*^No determination.
